# Evolution of the Xerocarpa clade (*Opuntia*; Opuntieae): Evidence for the Role of the Grand Canyon in the Biogeographic History of the Iconic Beavertail Cactus and Relatives

**DOI:** 10.3390/plants12142677

**Published:** 2023-07-18

**Authors:** Lucas C. Majure, Thomas H. Murphy, Matias Köhler, Raul Puente, Wendy C. Hodgson

**Affiliations:** 1 University of Florida Herbarium (FLAS), Florida Museum, Department of Natural History, University of Florida, Gainesville, FL 32611, USA; tmurphy1@ufl.edu; 2 Department of Research and Conservation, Desert Botanical Garden, Phoenix, AZ 85008, USA; rpuente@dbg.org (R.P.); whodgson@dbg.org (W.C.H.); 3 Department of Biology, University of Florida, Gainesville, FL 32611, USA; 4 Centro de Ciências Humanas e Biológicas, Departamento de Biologia, Universidade Federal de São Carlos (UFSCar), Sorocaba 18052-780, SP, Brazil; matias.k@ufrgs.br

**Keywords:** Colorado Plateau, Mojave Desert, *Opuntia*, prickly pears, Sonoran Desert

## Abstract

The formation of the western North American drylands has led to the evolution of an astounding diversity of species well adapted for such communities. Complex historical patterns often underlie the modern distribution of the flora and fauna of these areas. We investigated the biogeography of a group of desert-adapted prickly pears, known as the Xerocarpa clade, from western North America. The Xerocarpa clade originated in the mid-late Pliocene, likely on the Colorado Plateau, and then moved south into the Mojave, Sonoran, and Chihuahuan deserts, and California montane regions, further diversifying, mostly into the Quaternary. The southward trajectory of the clade was likely greatly influenced by the formation of the Grand Canyon. The synapomorphy of dry fruit presumably impeded the long-distance dispersibility of the beavertail cactus, *Opuntia basilaris*, while dry, spiny fruit may have enabled *O. polyacantha* to substantially increase its distribution. *Opuntia basilaris* evolved a pubescent epidermis, allowing it to invade hotter, drier conditions, while the spine-clothed stems of *O. polyacantha* may have given it an advantage for increasing its northern range into colder environments. The Xerocarpa clade shows a cold desert origin, and changes in morphological characters have made these sister taxa well adapted for invading broadscale, but oftentimes contrasting habitats.

## 1. Introduction

The evolution of the western North American dryland flora, including the timing and origin of species assemblages, is complex. One major factor limiting our understanding of the evolution of the flora is the lack of a consensus regarding the formation of western North American desert systems [1], although the initiation of these drylands at least dates back to the Miocene, coincident with the uplift of the Rocky Mountain system [2] and associated rain shadow effects [3]. Phylogenetic studies and fossil records of various organismal groups from the western North American deserts have provided further evidence for the timing of the development of these systems, which were more completely formed later into the Plio- and Pleistocene [4], where presumably desert-adapted taxa would have necessarily occurred in a well-formed dryland system. The origin of arid-adapted taxa may have been uniquely within western North American drylands, from where they further expanded their distributions outside of the area [5,6,7,8], or the expansion may have followed a south- to northward migration (e.g., out of South America), as has commonly been detected for several lineages of plants [9,10,11,12,13]. 

Cactaceae are a ubiquitous component of the western North American flora and, given their morphological adaptations for life in edaphically dry environments [14], provide an intriguing model to study the evolution of desert flora. It is clear from previous work that western North American cacti were derived from southern ancestors in several waves of migration from south to north, in some cases, presumably resulting from long-distance dispersal from distinct parts of South America [9,10,15,16,17]. This happened over a relatively short time frame, given that the family itself is likely geologically very young [15,16,17,18,19]. The prickly pear cacti or nopales of the genus *Opuntia*, are significantly much younger, with an estimated stem age of around 7.5 mya [15,19]. Thus, they provide an intriguing window into Plio-Pleistocene-related geological events and diversification in arid areas of the Americas.

Recent phylogenetic analyses have shed light on the broader relationships among major clades of the prickly pear cacti [9,20,21]. Based on data by Majure et al. [9], it was inferred that *Opuntia* s.str. evolved in southern South America during the Pliocene and then eventually dispersed into the North American desert regions, where the clade greatly expanded in alpha diversity, as well as range, now being distributed throughout the Americas from Patagonia to Canada, including throughout the Caribbean Islands and almost all of eastern North America [22]. Eight major clades were resolved in those previous phylogenetic analyses, two of those being strictly South American clades and the other six clades (i.e., the North American clades) mostly composed of North American taxa. Certain species are very widely distributed and, although sometimes found in South America, were derived from North American taxa and/or clades (e.g., *O. ficus-indica*, *O. pubescens, O. stricta* s.l.). The North American *Opuntia* clade most likely originated in parts of the Chihuahuan Desert and outlying areas [9], and thus was derived from tropical taxa or at least warm desert ancestors, a pattern strikingly similar to tribe Cacteae [17]. 

The Basilares clade, one of the subclades of the North American clade, is composed of the Excelsa, Microdasys, Rhizomatosa, and Xerocarpa subclades, based on previous work [9]. These clades are composed of taxa ranging from the Chihuahuan Desert to the Colorado Plateau and Baja California, and present wide variation in morphological features, from spineless, pubescent stems to glabrous, spiny stems. These may be small geophytic shrubs with massive rhizomes, such as in *O. pachyrrhiza,* or large, erect shrubs to small trees, as in *O. rufida* and *O. excelsa*, respectively [21,23,24], and they may have fleshy or dry fruit. 

The Xerocarpa clade, so termed for the production of dry fruit [9,25], consists of some of the most widespread species of prickly pear cacti in North America [e.g., *O. fragilis* (Nutt.) Haw., *O. polyacantha*] and is composed of two clades, the Polyacantha and Basilaris clades. The Basilaris clade consists mostly of diploid taxa, which are restricted to the Colorado Plateau, Sonoran, and Mojave deserts, as well as a few other minor ecoregions (and one diploid/triploid taxon, *O. basilaris* var. *treleasii*; [26]), whereas the Polyacantha clade consists of diploids, tetraploids, hexaploids, and octoploids [27], and is widespread from the northern Chihuahuan Desert north into parts of southern Canada. However, diploids of the *O. polyacantha* clade are restricted to western Texas, New Mexico, northern Mexico in parts of the Chihuahuan Desert [28,29], northeastern/northwestern Arizona–southeastern Utah, and parts of the Colorado Plateau and Mojave Desert [27,30,31,32]. az

**Figure 1:**
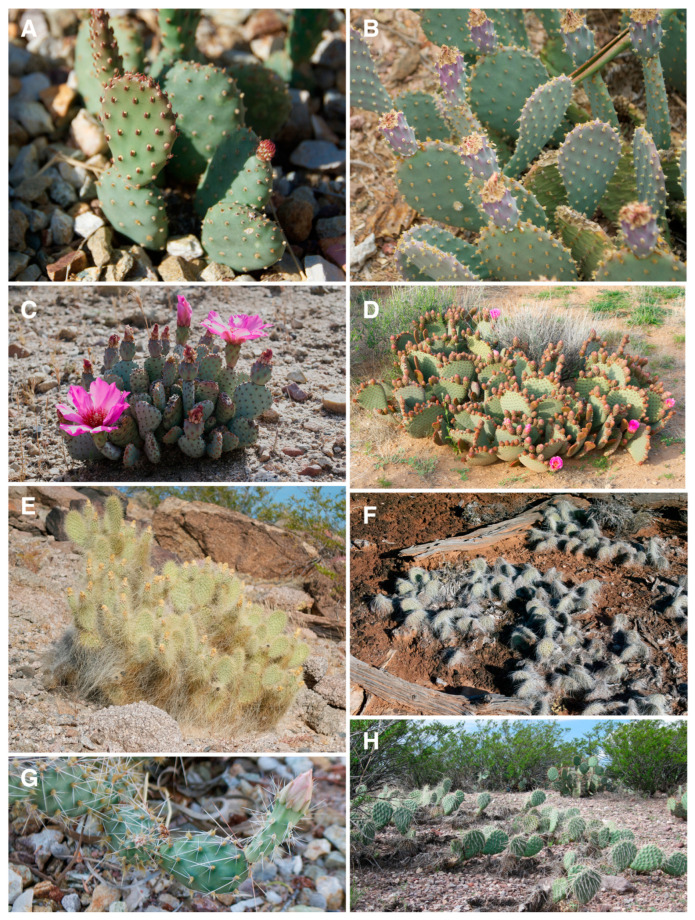
Plate of members of the *Xerocarpa* clade. (**A**) *Opuntia basilaris* var. *heilii* (Wayne Co., UT; cult. in Phoenix, AZ; *Majure 6067*), (**B**) *Opuntia basilaris* var. *longiareolata* (cult. in Phoenix, AZ; DBG 1952), (**C**) *Opuntia basilaris* var. *brachyclada* (San Bernardino Co., CA; *Majure 5747*), (**D**) *Opuntia basilaris* var. *basilaris* (Mohave Co., AZ; *Majure 5437*), (**E**) *O. diploursina* (Mohave Co., AZ; *Majure 5686*), (**F**) *O. polyacantha* var. *trichophora* (San Juan Co., UT; *Majure 6079*). (**G**) *O. polyacantha* var. *arenaria* (Cult., Phoenix, AZ; *Moore 2911*), (**H**) *O. polyacantha* var. *trichophora* (Socorro Co., NM; *Majure 3525*). Photos taken by L.C. Majure.

The Basilaris clade is composed of what has traditionally been considered one widespread species, *O. basilaris* s.l. (Figure 1A–D), which consists of four to seven infraspecific taxa [30]. Pinkava [30] recognized four varieties of *O. basilaris*: the widespread *O. basilaris* var. *basilaris* (2*n* = 22; Figure 1D) from Arizona (AZ), California (CA), Nevada (NV), Utah (UT), and parts of northern Mexico (MX); *O. basilaris* var. *brachyclada* (2*n* = 22; Figure 1C) from southwestern CA in the San Bernardino Mts; *O. basilaris* var. *longiareolata* (2*n* = 22; Figure 1B) from the Grand Canyon region; and *O. basilaris* var. *treleasii* (2*n* = 22, 33) from southeastern CA [30]. Several other varieties of *O. basilaris* also have been recognized by some, including *O. basilaris* var. *heilii* (Figure 1A) from southeastern UT [33], *O. basilaris* var. *ramosa* from western California [34], *O. basilaris* var. *whitneyana* from the Mt. Whitney area in southern California, and *O. basilaris* var. *woodburyi* (2*n* = 88) from Washington Co., UT. Pinkava (2003) considered *O. basilaris* var. *heilii* to be synonymous with *O. basilaris* var. *longiareolata* and *O. basilaris* var. *ramosa* to be synonymous with *O. basilaris* var. *brachyclada*, and Parfitt [27,35] and Pinkava [30] included *O. basilaris* var. *woodburyi* within his concept of the octoploid *O. pinkavae* in the *O. polyacantha* species complex (see below: Polyacantha clade). 

The Polyacantha clade (Figure 1E–H, diploids only) is composed of as many as nine species or as few as four species [27,30]. Parfitt (1991) considered *O. polyacantha* s.l., which he termed the *O. polyacantha* complex, to be one widespread species with five varieties, including *O. polyacantha* var. *arenaria* (2*n* = 22; Figure 1G), *O. polyacantha* var. *erinacea* (2*n* = 44), *O. polyacantha* var. *hystricina* (2*n* = 66), *O. polyacantha* var. *nicholii* (2*n* = 66), and *O. polyacantha* var. *polyacantha* (2*n* = 44). He also recognized *O. aurea* (2*n* = 66)*, O. fragilis* (2*n* = 66), and *O. pinkavae* (2*n* = 88) within the Polyacantha species complex, and it is evident that at least two of those polyploid taxa of the Polyacantha complex (e.g., *O. aurea, O. pinkavae*) are derived from hybridization with other clades [9]. Powell and Weedin [28] recognized another diploid variety of *O. polyacantha*, *O. polyacantha* var. *trichophora*. Breslin et al. [36] and Stock et al. [31] also consider the diploid populations of *O. polyacantha* in southeastern Utah and northeastern Arizona (Figure 1F) to be disjunct populations of *O. polyacantha* var. *trichophora* (as *O. trichopora* in their work), which is mostly found in southwestern Texas and southeastern-central New Mexico [28] (Figure 1H). Recently, Stock et al. [31] have recognized another species from the group, *O. diploursina* (2*n* = 22; Figure 1E), which previously had been considered synonymous with the tetraploid *O. polyacantha* var. *erinacea* [27]. Stock et al. [31,33] also accept most infraspecific taxa, sensu Parfitt [27], as species, apparently based mostly on differences in ploidy and some cryptic morphological characters.

Intriguingly, the Polyacantha and Basilaris clades had not been considered close relatives by Parfitt [27] or Pinkava [30,37], presumably based on their seemingly divergent morphologies. However, Majure et al. [9] showed that the two clades were sisters, of relatively old age (Pliocene in origin), and shared the putative synapomorphy of dry fruit. Both the Basilaris and Polyacantha clade members produce stem segments that are densely covered in areoles, but the Basilaris clade is essentially spineless (Figure 1A–D), while the Polyacantha clade, in contrast, represents one of the spiniest groups of *Opuntia,* their epidermis oftentimes totally obscured by spines (Figure 1E–H). This contrasting morphology is of further interest given that diploids of the two clades sometimes grow together (*O. basilaris* and *O. diploursina* are sympatric at Lake Mead, AZ and NV); thus, these taxa appear to have different strategies for growing in similar environments under the same climatic and ecological conditions. Fascinating populations of the *O. basilaris* var. *heilii* taxon (often considered synonymous with *O. basilaris* var. *longiareolata* [30]) occur in southern Utah, and these populations have a glabrous epidermis, in contrast to most other populations of *O. basilaris*. Likewise, some collections of *O. basilaris* var. *longiareolata* do not exhibit extensive pubescence, but rather minor papillae on the epidermis (or are even sometimes mostly glabrous), thus potentially showing morphological intermediacy between *O. basilaris* var. *heilii* and other members of *O. basilaris,* as well as the glabrous members of the Polyacantha clade. But morphological evolution in this group has never been reconstructed, and this entire group of diploid taxa has never been analyzed phylogenetically. 

It is curious that the diploids in the Xerocarpa clade are restricted to both cold (Colorado Plateau) and warm (Chihuahuan, Mojave, and Sonoran) deserts, although essentially all other North American Opuntias appear to be derived from warm deserts (i.e., Chihuahuan Desert) or tropical areas, such as seasonally dry tropical forests (e.g., Nopalea clade sensu Majure et al. [9]). Pinkava [37] hypothesized that the *O. basilaris* complex likely originated on the Colorado Plateau, while the *O. polyacantha* complex originated further south in the Chihuahuan Desert in Mexico, this idea owed mostly to their current distribution. In situ radiations within western North American drylands are poorly studied, and this is especially the case for taxa in and around the Grand Canyon region of the Colorado Plateau, a major geological feature that formed over the past nearly 20 million years (mya; [38]), but with most erosional depth occurring presumably within the last 5–6 mya [39]. Given the curious northern distribution of some of the diploid taxa in the Basilaris and Polyacantha sister clades, the potential geographic origin for this fascinating and apparently relatively old clade, comparatively [9], may likely have been in and around the Grand Canyon region of the Colorado Plateau (i.e., a cold desert origin). 

Here we expanded sampling of the diploids of the Xerocarpa clade to test our hypothesis that the Xerocarpa clade originated in the cold desert system of the Colorado Plateau and then migrated south, a very different pattern than that seen in other North American prickly pears, which appear to have moved north out of the Chihuahuan Desert. We reconstructed the phylogeny of the group using plastome data derived from genome skimming to test the resolving power both among and within species of the Xerocarpa clade. We then reconstructed the biogeographic history of the Xerocarpa clade across western North America and analyzed the evolution of salient morphological features in the clade. 

## 2. Results

### 2.1. Sequencing, Assembly and Alignments

Raw reads from genome skimming yielded between 5.7–18.5 million reads, and roughly 2.6–9.1% of the reads were plastid, based on our reference-guided assembly using our partially assembled plastome of *O. basilaris*. The assembly of *Opuntia basilaris* (*Majure 5753*) yielded a chloroplast genome of 125,000 bp that lacked one copy of the inverted repeat and also exhibited a ca. 6000 bp inversion of the *atpB-atbE* gene suit, as was shown for saguaro by Sanderson et al. [40] and other cacti [10,41]. Our concatenated alignment of the three major regions of the *Opuntia* chloroplast genome (i.e., IR, LSC, SSC) yielded an alignment 127,534 bp long. 

### 2.2. Phylogeny

**Figure 2:**
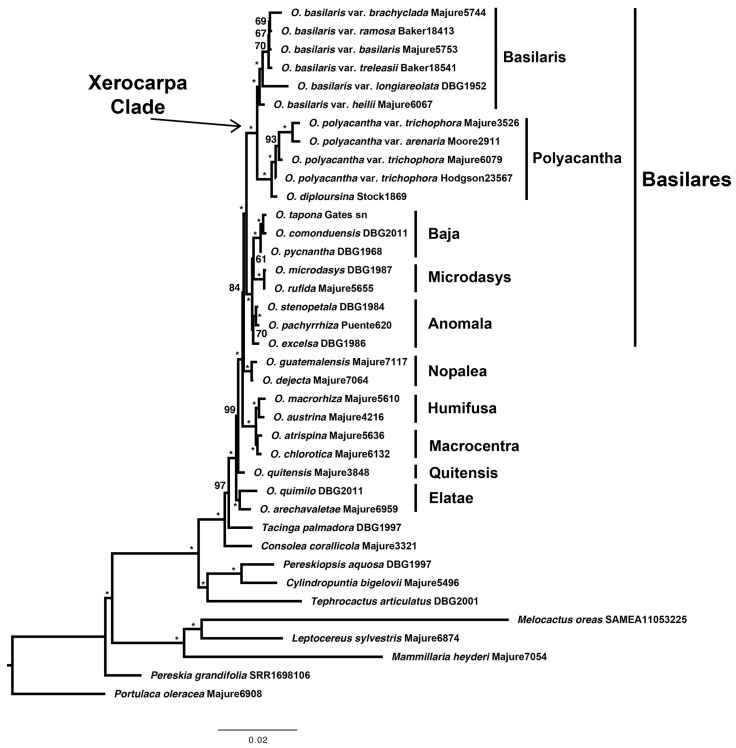
Phylogeny of the Xerocarpa clade based on plastomes assembled from genome-skimming data. The Xerocarpa clade was sister to the clade composed of the Baja, Microdasys, and Anomala clades, all of which make up the Basilares clade. The Basilaris and Polyacantha clades were well supported (bs = 100). *Opuntia basilaris* var. *heilii* was sister to the Basilaris clade, and *O. diploursina* was sister to the Polyacantha clade. Bootstrap values are given at the nodes; asterisks are given for bootstraps of 100 and are written out for values < 100.

The tribe Opuntieae was well supported (bs = 100), with *Consolea corallicola* recovered as sister to the rest of the clade. The Elatae clade was sister to the rest of *Opuntia*, and the Quitensis clade was sister to the North American clade. The Macrocentra and Humifusa clades were sister to the Nopalea + Basilares clade. Nopalea clade members *O. dejecta* and *O. guatemalensis* were well supported (bs = 100) as sister to the Basilares clade, and the Microdasys (*O. microdasys + O. rufida*), Baja (*O. pycnantha* + (*O. comonduensis + O. tapona*), and *O. excelsa* + (*O. pachyrrhiza* + *O. stenopetala*) clade, here referred to as the Anomala clade, formed a well-supported clade (bs = 100), which was sister to the well-supported Xerocarpa clade (bs = 100, containing the Basilaris and Polyacantha clades). The Basilaris clade was well supported (bs = 100) and sister to the well-supported Polyacantha clade (bs = 100, Figure 2). 

*Opuntia basilaris* var. *heilii* from the Colorado Plateau was resolved as sister to all other members of the *O. basilaris* clade, followed by *O. basilaris* var. *longiareolata* from in and around the Grand Canyon, which was resolved as sister to the rest of the members of the clade (i.e., from the Mojave/Sonoran deserts). *Opuntia basilaris* var. *treleasii* was sister to a clade containing *O. basilaris* var. *basilaris, O. basilaris* var. *ramosa*, and *O. basilaris* var. *brachyclada*. Although infraspecific relationships were resolved in the *O. basilaris* var. *treleasii* + *O. basilaris* var. *brachyclada* subclade, bootstrap support was low (bs = 70 and below). *Opuntia diploursina* was resolved as sister to the rest of the members of the *O. polyacantha* clade. *Opuntia polyacantha* s.l. from NE AZ/SE UT was resolved in a grade sister to material from the Chihuahuan Desert (*O. polyacantha* var. *arenaria* and *O. polyacantha* var. *trichophora*). Relationships among terminals within the Polyacantha clade were well supported (bs = 93–100).

### 2.3. Divergence Time Estimation

**Figure 3:**
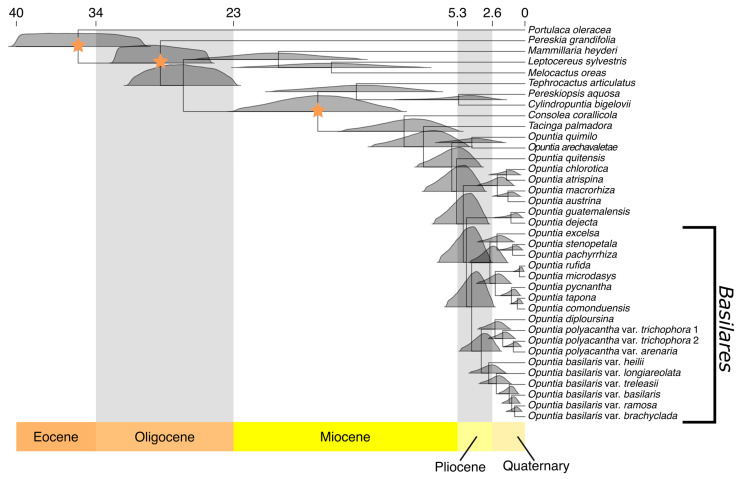
Time-calibrated phylogeny of the *Basilares* clade with representative sampling of major clades of Cactaceae and its outgroup. Stars indicate the three nodes that were constrained based on secondary calibrations from [19] of the stem of Cactaceae, crown of Cactaceae, and crown of Opuntioideae. Posterior distributions of node ages are shown by density plots at each node, centered on the mean age. Numbers at the top of the figure represent millions of years. Most clades of Opuntieae had a Pliocene origin, as did the Basilares clade, which arose in the Mid Pliocene. The Xerocarpa clade had a putative mid-late Pliocene origin. The Basilaris clade also had a late Pliocene origin, although most lineages were derived within the Pleistocene. The Polyacantha clade arose during the early Pleistocene.

Recovered divergence time estimates for the *Basilares* clade were 4.62 MYA (2.91–6.91 MYA, 95% highest probability density [HPD]) for the stem and 4.21 MYA (2.63–6.31 MYA, 95% HPD) for the crown, both of which were of Pliocene to Late Miocene age. The crown of the *Xerocarpa* clade was recovered as Early Pleistocene to Late Pliocene in age, 3.45 MA (2.13–5.25 MYA, 95% HPD). The other major subclade in the *Basilares* clade, which contains taxa of the subclades Baja, Anomala, and Microdasys, had a mean crown age of 2.77 MYA (1.57–4.49 MYA 95% HPD), dating from the Middle Pliocene to Early Pleistocene (Figure 3).

### 2.4. Ancestral Area Estimation

**Figure 4:**
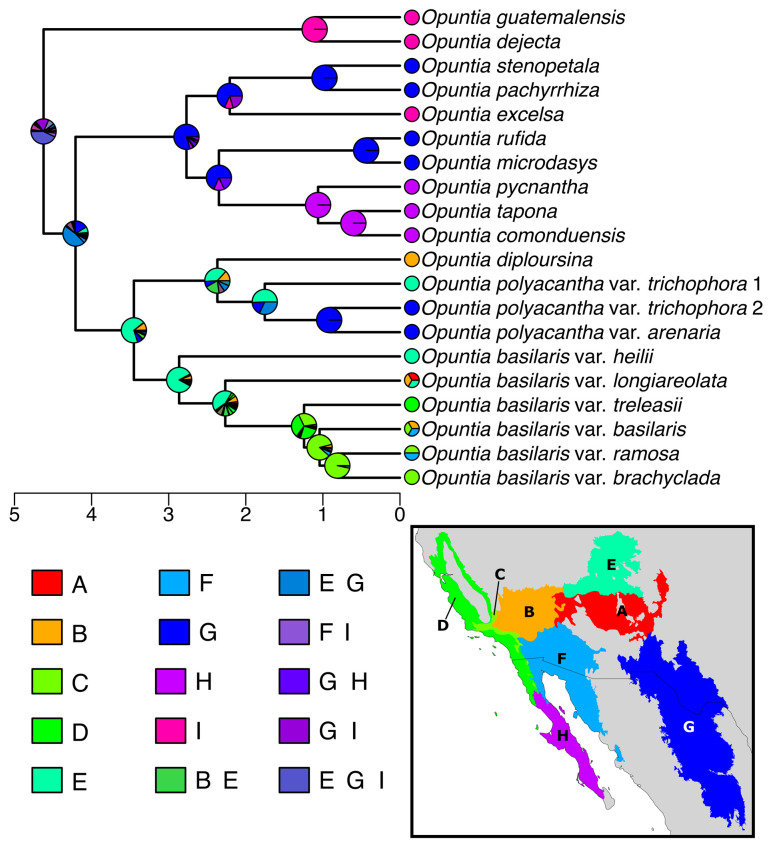
Ancestral area reconstruction of the Xerocarpa clade based on biogeographic analysis with the DIVALIKE+J model. Pie charts at nodes depict probabilities of ancestral ranges, and coding for each terminal is given as a pie chart. Area states recovered in the top two probabilities for at least one node or representing a single area are shown in the legend. Areas are as follows: (A) AZ–NM Plateau, (B) Mojave Desert, (C) CA Pine Oak Mts, (D) CA Coastal Sage/Oak Woodlands, (E) Colorado Plateau, (F) Sonoran Desert, (G) Chihuahuan Desert, (H) Baja California, and (I) seasonally dry tropical forest of Central America and Mexico (SDTF; not shown). Scale below the phylogeny depicts millions of years based on the divergence time estimation analysis. The Basilares clade was reconstructed as having a Chihuahuan Desert–Colorado Plaeau origin, while the Anomala + Baja + Microdasys clade was Chihuahuan (G) in origin with a movement into Baja California (H) in the Baja clade. The Xerocarpa clade originated in the Colorado Plateau (E) and then returned to the Chihuahuan Desert (G) in the Polyacantha clade, and the Basilaris clade migrated south into the AZ–NM Plateau (A), the Mojave (B) and Sonoran (F) deserts, and finally into the CA mountain ecoregions (C,D).

Of the six models tested (Table 1), DIVALIKE+J had the highest log-likelihood (−49.65643) and lowest AICc (106.8129). We do note, however, that it was only marginally better-fit than DEC+J based on both log-likelihood (−50.40807) and AICc (108.3161). To avoid overinterpretation of results from a single analysis, we examined the ancestral area probabilities from DEC+J but focus our discussion here on the DIVALIKE+J analysis (Figure 4). Results from DEC+J are included in Supplementary Figure S1.

The *Basilares* clade was inferred to have a Chihuahuan Desert + Colorado Plateau origin (probability [Pr] = 0.47), while the *Xerocarpa* subclade was found to have a Colorado Plateau origin (Pr = 0.69). *Opuntia basilaris* was strongly supported as having a Colorado Plateau origin with a clear transition south into the AZ–NM Plateau, the Mojave Desert, and then into the Sonoran Desert, the CA Pine Oak Mts, and CA Coastal Sage/Oak Woodlands. The Polyacantha clade was supported as having a Colorado Plateau origin (Pr = 0.38) with support for subsequent transition to the Chihuahuan Desert (Pr = 1.00). The origin of the *Excelsa* + *Rhizomatosa* + *Microdasys* subclade was recovered in the Chihuahuan Desert (Pr = 0.80). The clade containing *O. pycnantha*, *O. tapona*, and *O. comonduensis* was inferred as having an origin in the Baja California Desert (Pr = 1.0; Figure 4).

### 2.5. Morphological Evolution

**Figure 5:**
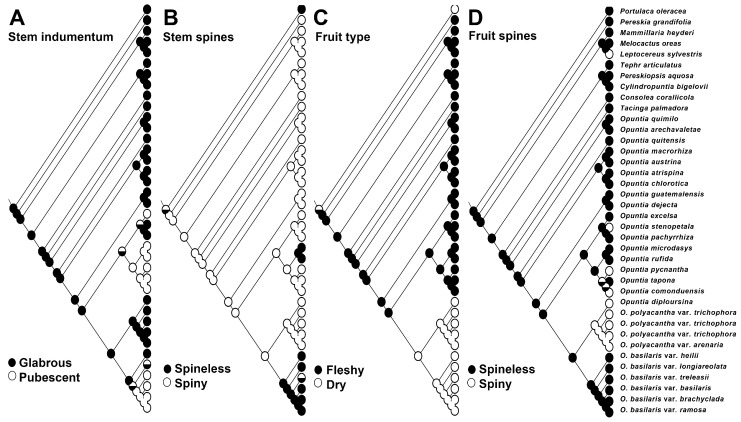
Morphological evolution in the Xerocarpa clade. (**A**) Pubescent stems evolved at least twice in the Basilares clade, once within the Basilaris clade and perhaps once or twice within the Anomala + Baja clade. (**B**) Spiny stems were plesiomorphic and the loss of spines was synapomorphic for the Basilaris and Microdasys clades. (**C**) Dry fruit were a synapomorphy of the Xerocarpa clade. (**D**) Spiny fruit were synapomorphic for the Polyacantha clade and likewise evolved elsewhere in the Anomala and Baja clades.

Glabrous, spiny stems were ancestral for the Xerocarpa clade. The sister to the rest of the *O. basilaris* clade, *O. basilaris* var. *heilii*, showed plesiomorphy in the presence of indumentum with glabrous stems, and *O. basilaris* var. *longiareolata* was polymorphic for this state, while the rest of the clade shared the derived state of having pubescent stems. Non-spiny stems also were a synapomorphy for the *O. basilaris* clade, although *O. basilaris* var. *treleasii* was polymorphic for this character, showing both spiny and non-spiny stems. Spineless stems were also synapomorphic for the Microdasys clade. Pubescent stems were equivocal for the Anomala + Baja + Microdasys clade, although ACCTRAN (accelerated transformation) optimization, if implemented, would show pubescent stems to be synapomorphic for the clade. Dry fruit were a clear synapomorphy for the entire Xerocarpa clade, as fleshy fruit were plesiomorphic for *Opuntia*, and spiny fruit were synapomorphic for the *O. polyacantha* clade. Spineless fruit were plesiomorphic, although spiny fruit were also found in *O. comonduensis* and *O. pycnantha* of the Baja clade, and *O. stenopetala* of the Anomala clade (Figure 5).

## 3. Discussion

### 3.1. Phylogeny

The phylogenetic topology recovered here is similar to that of other findings [9]. However, relationships within the Basilares clade are different from those obtained in Majure et al. [9] in several ways. Majure et al. [9] recovered *O. stenopetala* nested within the Microdasys clade, where here the species was sister to *O. pachyrrhiza*. *Opuntia excelsa* was poorly supported (bs = 57) as sister to the Baja clade in Majure et al. [9], where here it was sister to the *O. stenopetala* + *O. pachyrrhiza* clade, although that position was still poorly supported (bs = 70). Non-overlapping taxon sampling and Sanger (six plastid and two nuclear markers in Majure et al. [9]) versus plastome datasets likely are responsible for these topological differences.

Our topology here also differed in several ways from that of Majure et al. [15], which was based solely on chloroplast genes from across the plastome. Majure et al. [15] recovered the Quitensis clade as sister to the Elatae + North American clade, and the Nopalea clade was sister to the rest of the North American clade, rather than to the Basilares clade, which in Majure et al. [15] was sister to the Scheeri + (Macrocentra + Humifusa) clade. Köhler et al. [41] has already shown that different combinations of plastid genes can result in differing phylogenetic topologies. Perhaps the topological differences between Majure et al. [15] and our current dataset are a direct result of using an almost entire plastome here versus just genes in [15]. Differences between our current dataset and that of Majure et al. [9] could be a result of the combined use of plastid and nuclear loci for their work, as well as a lack of resolution in those previous analyses. Lastly, topological incongruences could also potentially result from non-overlapping taxon sampling: all three topologies had slightly different taxon sampling. Regardless, the topological differences should not affect the interpretation of our results here, given that the Basilares clade is still resolved and supported, and the Microdasys + Anomala + Baja clade is still resolved as sister to the Xerocarpa clade. Future work in *Opuntia* will need to address topological differences in plastome-level datasets and be compared with robust nuclear datasets.

### 3.2. Biogeographic Patterns

The split Colorado Plateau/Chihuauhuan Desert origin of the Basilares clade provides a logical explanation for the origin and expansion of this clade into cold desert systems. As was proposed by Majure et al. [9], it appears most likely that the North American *Opuntia* clade had an origin in and around the Chihuahuan Desert. Logically, taxa would have subsequently continued to move norward into the Colorado Plateau, northeasterly into eastern North American forests, or northwesterly into the Sonoran Desert, given appropriate environmental conditions. Thus, it seems likely that ancestors of the Basilares clade could have ended up in the Colorado Plateau through northward migration in the mid-early Pliocene and adapted to local conditions leading to the in situ evolution of the Xerocarpa clade, with more southerly ancestral populations going extinct. The well-adapted Xerocarpa clade then would have begun its march southward from the Colorado Plateau. Other workers have suggested that the Colorado Plateau could have acted as a refugium given it was more climatically stable during parts of the Pleistocene, while areas outside of the plateau, such as the Mojave Desert, were less climatically stable [42,43]. This could perhaps help explain the pattern seen here, although on a much longer timescale.

Pinkava [37] described the origin and spread of *O. basilaris* and varieties from northeast to southwest (i.e., from the sands of southeastern Utah to the mountains and lower desert regions of the Sonoran Desert and southern California mountains). This hypothesis of origin and spread of the *O. basilaris* complex is congruent with our phylogenetic hypothesis of relationships in the group and results from our biogeographic analyses. It appears most likely that the Basilaris clade originated along the upper reaches of the Grand Canyon biogeographic region of the Colorado Plateau and then spread south and west into the Mojave and Sonoran Deserts, and eventually into the CA Oak Pine Mts and CA Coastal Sage/Oak Woodlands.

On the contrary, Pinkava [37] suggested that the *O. polyacantha* group originated in Mexico, south of most of its current distribution, and then subsequently spread north. Based on our data, it appears most likely that the Polyacantha clade originated, like the *O. basilaris* clade, in the Colorado Plateau, from where it spread south into the Chihuahuan and Mojave deserts. The disjunct distributions as seen in populations of *O. polycantha* var. *trichophora*, from SE UT/NE AZ and then to western TX and eastern NM, could have been produced through a southern migration of *O. polyacantha* with subsequent extinction of intermittent populations between the two areas. Phylogeographic studies of several groups of plants suggest that the expansion of favorable habitat during Pleistocene glacial events led to the population expansion of certain species, such as *Canotia holacantha*, which subsequently went extinct, leaving behind refugial populations of those species with appropriate ecologies [44].

The putative southward migration from the Colorado Plateau in the Basilaris and Polyacantha clades is further evidenced by the distribution of diploids and polyploids in those clades, as compared with other species complexes of *Opuntia*. Majure et al. [45] showed that diploids in the Humifusa complex occupied more southerly limits of the distribution of that clade, while polyploids mostly formed around diploid populations and moved further north into previously glaciated parts of their current range. Diploid members of the Macrocentra clade, such as *O. macrocentra*, are found further south in the Chihuahuan Desert, for example in the Big Bend area of southern Texas, while polyploid derivatives are found further north around Alpine, Texas and then north into New Mexico [28,37]. Diploid relatives of the *O. engelmannii* complex (e.g., *O. cuija/cantabrigiensis*) are found in the states of Querétaro and San Luís Potosí, Mexico, while more northern distributions of close relatives mostly consist of hexaploids, for instance in western United States populations of *O. engelmannii* s.l., thus, showing a strikingly similar pattern of northward migration and polyploid formation.

Extending out from diploid populations of the Polyacantha clade, polyploid components of the distribution are found to the east, west and mostly to the north of those areas, reaching as far as Saskatchewan, Canada. The Polyacantha clade also barely reaches northern Mexico, where *O. polyacantha* var. *arenaria* occurs in the state of Chihuahua [27], suggesting that the clade did not originate from further south based on common patterns seen with polyploid complexes in *Opuntia*. Likewise, that the morphologically distinct *O. polycantha* var. *arenaria* is nested within a more homogeneous and paraphyletic *O. polyacantha* var. *trichophora* suggests perhaps the adaptation to significantly different ecological constraints, a pattern also akin to peripheral isolate speciation [46]. Thus, the Polyacantha clade follows a very similar cytogeographic pattern as compared to other prickly pear species complexes and mirrors the *O. humifusa* species complex, with most of the distribution to the north of the putative center of origin, where diploids still remain greatly restricted [22,45]. *Opuntia polyacantha* var. *hystricina* was recorded from the Sierra de Mapimí [47]; however, that material appears to be more closely related to the Humifusa clade, specifically the *O. macrorhiza*/*O. pottsii* species complex, given its spineless fruit. Thus, that material was not considered as part of the distribution of *O. polycantha* here.

The formation of the Colorado River, and subsequently the Grand Canyon, provides another line of evidence supporting the southward migration in both the Basilaris and Polyacantha clades. The putative origin of the Grand Canyon dates from 5–6 mya with the linking of Marble Canyon and the Westernmost Grand Canyon with two other major segments that formed the contemporary Colorado River [48], which formed the canyon through erosion to current depths mostly over the past 4 mya [39]. Considering the putative age of the Xerocarpa clade at <4 mya based on our divergence time estimation and the possession of dry fruit that are mostly dispersed over short distances by rodents (e.g., [49]), the clade would likely have been greatly impeded by the formation of the Grand Canyon, which at that point would have been a substantial barrier to dispersal. However, given the potential for seed or stem dispersal by water [50,51] and that the northernmost populations of *O. basilaris* (*O. basilaris* var. *heilii*) essentially occurring north of the Grand Canyon in the headwaters of a tributary of the Colorado River, the Dirty Devil River, a southward migration via water courses could have been a logical path. This would also send propagules of *O. basilaris* onto the eastern or southernmost edges of the Colorado River, leading to the skewed southern/eastern distribution as seen today. *Opuntia basilaris* var. *longiareolata*, the taxon that is confined to the Grand Canyon area, is mostly restricted to the southern and eastern rim of the Grand Canyon. Indeed, there is evidence for lake formation, along with large-scale dam outbursts and downstream flooding, which resulted from a series of lava flows along the canyon over the past million years or so [52]. This certainly could have affected populations of *O. basilaris* along the canyon. On the contrary, populations of *O. basilaris* could have simply migrated south, along, and mostly restricted to, the eastern edge of the Grand Canyon, and populations in between current day *O. basilaris* var. *heilii* and *O. basilaris* var. *longiareolata* could have gone extinct. Perhaps the Grand Canyon also played a role in shaping the distribution of diploid *O. polyacantha* members, given that *O. polyacantha* var. *trichophora* is found east (and southeast) of the canyon [27,37], and *O. diploursina* is mostly confined to the southern edge of the canyon around Lake Mead [31]. Patterns seen in populations of *Coleogyne ramosissima* show similar disjunctions between the Colorado Plateau and the Mojave Desert [43].

### 3.3. Morphological Evolution

Taxa with pubescent stems often lack spiny stems, although this is not ubiquitous. *Opuntia comonduensis, O. pycnantha*, and *O. tapona* have pubescent, spiny stems, as do other species not sampled here, such as *O. durangensis* and *O. pubescens*. Stem pubescence, as in leaves, is thought to be an adaptation for reducing transporation rates and moderating stem temperatures, as do spines [53], which are necessary adaptations for existing in hotter, drier climates [15], such as theAZ–NM Plateau, Sonoran, and Mojave deserts. Gibson and Nobel [14] also mention the production of pubescence as an adaptation to colder temperatures in cacti, such as in northern populations of the genus *Ferocactus*. That does not appear to be the case in *Opuntia*, and in general, those taxa with pubescent stems occur in warmer environments, such as the Sonoran Desert and Baja California, in species sampled here (e.g., *O. basilaris* var. *basilaris*, *O. comonduensis*). Stem color exhibited by the *O. basilaris* clade (glaucous blue, maroon-purple) may also be an adaptation for reflecting sunlight and thus reducing stem overheating through betalain pigment presentation. The spiny stems of the *O. polyacantha* clade, although likely aiding in reflecting photosynthetically active radiation (PAR) and thus moderating high stem temperatures, perhaps also confer an advantage for existing in cooler climates. Spines have an insulating effect against colder temperatures [53,54] and even diploid *O. polyacantha* var. *trichophora* can easily withstand freezing temperatures under snow (Majure, pers. obsv.), this likely also as a result of reducing stem water content, i.e., hardening of the stems [55]. *Opuntia polyacantha* clade members have some of the largest distributions of all *Opuntia*, occurring in very cold environments. Stems covered in a dense network of spines may have acted as a preadaptation for expanding into these colder, more northerly habitats. Cold tolerance and even preference may also help explain their putative expansion during Pleistocene glacial events, as mentioned above.

Dry fruit, although not as readily dispersed over long distances from vertebrate consumption, could possess other advantages in desert environments [56]. Fleshy fruit would necessarily need more water resources to develop and be maintained through to maturity, while the thin pericarp of dry-fruited species should require fewer water resources for development. Dry fruits are lighter than juicy fruits and can disperse passively after falling off of the parent plant. This has been seen in the Mojave and Sonoran deserts and Colorado Plateau in members of the Xerocarpa clade, where the fruits are dispersed over short distances downslope through gravity or by strong winds (Majure, pers. obsv.). Fruits and seeds also can be carried some distance from parent plants by runoff from seasonal storms (R. Puente, pers. obsv.). Desert plants, especially those with bur-like fruit or fruit with winglike projections or long trichomes, have been shown to be easily dispersed by wind or anemochory [57]. However, it seems that dry fruits with relatively poor dispersal ability are common in desert plants [56].

Dry fruits, the seeds of which are commonly consumed and inadvertently dispersed by small mammals, such as rodents, would likely be more successful in hyper dry environments, where larger mammals may be less plentiful. Species producing dry fruit also seem to be more common in drier habitats [58,59]. The spines on the dry fruit of members of the *O. polyacantha* clade are generally strongly retrorsely barbed at the apex and easily stick into fur and skin of animals living among those populations; thus, epizoochory could have played a major role in their dispersal. Indeed, these fruits are referred to as bur-like by Pinkava [30]. This perhaps could help explain the very large distribution of the *O. polyacantha* clade, as contrasted with the more restricted distribution of the *O. basilaris* clade. Majure and Ribbens [60] conjectured that the modern distribution of *O. fragilis*, a member of the *O. polyacantha* clade, may have reached its large distribution through a similar mechanism, although directly through stem segments with retrorsely barbed spines being dispersed by migrating buffalo rather than via the dry fruit they seldom produce. Bur-like fruit also lodge more easily into surrounding vegetation and could potentially act as anchors under nurse plants for more ready establishment in water-limited, high-temperature/PAR environments (reviewed in [61]).

Some species of the chollas, *Cylindropuntia*, also produce spiny, dry fruit [10] much like those of *O. polyacantha* and are commonly consumed by packrats and other small rodents. *Opuntia basilaris, O. polyacantha*, and numerous species of *Cylindropuntia* have been found in Pleistocene–Holocene packrat middens (reviewed in [62]), although *Cylindropuntia* and *Opuntia* species in these middens were not restricted to just dry-fruited species [63]. Thus, short-distance (i.e., regional) dispersal by such small mammals must be taken into consideration when considering changes in distribution over long time periods.

### 3.4. Taxonomy in the Xerocarpa Clade

Although Pinkava [30] placed *Opuntia basilaris* var. *heilii* in synonymy with *O. basilaris* var. *longiareolata*, given the lack of gene flow among those populations, as shown here with these two taxa occurring in phylogenetically disparate clades, *O. basilaris* var. *heilii* deserves recognition. Minor morphological characters could be used to recognize these two taxa, with *O. basilaris* var. *heilii* having a glabrous epidermis and *O. basilaris* var. *longiareolata* having a mostly pubescent epidermis. The number of areoles per cladode face and epidermal color can be useful for distinguishing *O. basilaris* var. *heilii* from other members of the clade, generally [33]. Likewise, we consider the infraspecific rank of subspecies more acceptable considering the geographic cohesiveness and presumed lack of interbreeding among populations of the two taxa with their non-overlapping distributions [64]. We propose the following two combinations at the subspecies level.

***Opuntia basilaris* subsp. *heilii* (S.L.Welsh & Neese) Majure, stat. nov.** Basionym: *Opuntia basilaris* var. *heilii* S.L.Welsh & Neese Great Basin Naturalist 43: 700. 1984.

***Opuntia basilaris* subsp. *longiareolata* (Clover & Jotter) Majure, stat. nov.** Basionym: *Opuntia longiareolata* Clover & Jotter Bull. Torrey Bot. Club 68: 418. 1941. *Opuntia basilaris* var. *longiareolata* (Clover & Jotter) L.D.Benson Cacti Ariz. 43. 1950.

It is clear that *O. basilaris* var. *basilaris*, *O. basilaris* var. *brachyclada*, *O. basilaris* var. *ramosa*, and *O. basilaris* var. *treleasii* are more closely related to one another than they are to either *O. basilaris* var. *heilii* or *O. basilaris* var. *longiareolata*, but understanding fine-scale relationships among populations of those taxa is not possible with the current dataset. Thus, although we would suggest recognizing those taxa based on morphology, geographic distribution, and our phylogenetic results here, more refined population-level sampling with more robust datasets will be necessary to tease apart population-level relationships across the distribution of each of these taxa.

*Opuntia polyacantha* var. *trichophora* is clearly non-monophyletic and forms a grade with *O. polyacantha* var. *arenaria* nested within. Material of *O. polyacantha* var. *trichophora* from New Mexico (*Majure 3526*) was most closely related to *O. polyacantha* var. *arenaria*, from the same region, so there is a geographic signal to the relationships presented here. Material of *Majure 3526* is not typical of *O. polyacantha* var. *trichophora*, having much more robust spines, and thus could represent a separate taxon in need of recognition. However, the current dataset is not sufficient to tease apart such a taxonomically complicated situation and deserves much more detailed future work, including more deeply understanding the cytogeography across populations of *O. polyacantha* s.l. Considering the current topology, *O. polyacantha* var. *arenaria* essentially shows a pattern of peripheral isolate speciation [46] out of the more widespread *O. polyacantha* var. *trichophora*.

*Opuntia diploursina*, which is sister to the rest of the Polyacantha clade, seems to merit recognition at the species level, based both on phylogenetic position, as well as morphological differences compared with other diploid members from the clade. Stock et al. [31,32] consider *O. diploursina* and *O. polyacantha* var. *trichophora* (as *O. trichophora*) to be ancestral diploids to other polyploid members of the Polyacantha clade. Perhaps ancestral populations of those diploid taxa could have provided the diploid genomic components for what eventually resulted in polyploids in the *O. polyacantha* complex, such as for the tetraploids *O. erinacea* and *O. polyacantha* s.s. However, given that *O. diploursina* and *O. polyacantha* var. *trichophora* remain extant, and polyploids are presumably older than F1 generation hybrids, they should not be considered ancestral to the polyploids. Stock et al. [32] consider that *O. polyacantha* s.s. is an allotetraploid and thus should not be considered conspecific with other members of the clade, such as the diploids, *O. polyacantha* var. *arenaria*, and *O. polyacantha* var. *trichophora*. While an allotetraploid origin would suggest that *O. polyacantha* s.s. should not be considered conspecific with its diploid relatives, there are no clear data to suggest that *O. polyacantha* s.s. is indeed of allotetraploid origin, at least when taking the entirety of the populations into account. Based on morphological similarity among some populations of *O. polyacantha* s.s. and *O. polyacantha* var. *trichophora* [27,28,30,36], it does seem possible that some polyploid populations could be autopolyploids. Thus, the origin of polyploids across the *O. polyacantha* clade and taxon delimitation appears to be much more complicated than that proposed by Stock et al. [32], and the Polyacantha clade does indeed overlap in characters in terms of what would be considered a species complex [64]. Thus, we refrain from making any broadscale conclusions regarding species circumscriptions in the clade until more comprehensive chromosomal, morphological, and phylogeographic datasets at the population level across the distribution of the clade are generated.

## 4. Materials and Methods

### 4.1. Taxon Sampling

We sampled all known diploid members of the *Basilares* clade (sensu [9]), including all of the currently recognized diploid taxa of the Xerocarpa clade (i.e., Basilaris and Polyacantha clades). The northernmost *O. basilaris* populations were sampled from UT (*O. basilaris* var. *heilii*), along the Grand Canyon (*O. basilaris* var. *longiareolata*), the San Bernardino Mts (*O. basilaris* var. *brachyclada*), the mountains of southwestern California (*O. basilaris* var. *ramosa*), south-central California (*O. basilaris* var. *treleasii*), and part of the distribution of *O. basilaris* var. *basilaris* from the Mojave/Sonoran deserts. Diploid *O. polyacantha* s.l. (i.e., *O. polyacantha* var. *trichophora*) was sampled both from the NE AZ/SE UT populations, as well as SW New Mexico (*O. polyacantha* var. *trichophora*) and adjacent west TX (*O. polyacantha* var. *arenaria*) (see Table 1), and the recently described taxon, *O. diploursina,* was sampled from the Lake Mead area in Mohave Co., AZ. All other diploid members of the *Basilares* clade (*Excelsa, Microdasys, Rhizomatosa* clades) were sampled, and outgroups consisted of the Nopalea, Humifusa, Macrocentra, Quitensis, and Elatae clades of *Opuntia*, as well as *Tacinga palmadora, Consolea corallicola* (both tribe Opuntieae), Cylindropuntieae, Tephrocacteae, Cactoideae, *Pereskia grandifolia*, and *Portulaca oleracea*, based on data from previous studies [15] (see Appendix A).

### 4.2. DNA Extraction, Sequencing, Alignment, and Phylogenetic Analysis

DNA extraction was carried out using a modified CTAB extraction buffer [65] on either fresh, silica-dried tissue or herbarium samples. A genome-skimming approach [66] with the goal of sequencing nearly entire plastomes for phylogenetic analyses was carried out. Those selected samples were extracted using a modified CTAB protocol, but the supernatant was purified with silica columns using the methods of [10,67].

Samples used for genome skimming were sent to Rapid Genomics for library building and were sequenced on an Illumina HiSeq X Ten platform yielding 150 bp paired-end reads. A select number of samples were assembled using velvet [68] in the Galaxy platform (https://galaxy.rc.ufl.edu, accessed on 12 June 2023) after pairing reads with FASTQ Interlacer and quality control using FASTQ Quality Trimmer [69] with a window size of 5 and quality score of 20. The most complete assembly (*O. basilaris*; *Majure 5753*) was used for reference-guided assembly of the other sequenced samples in Geneious (Biomatters, LLC.). As the chloroplast genome in Cactaceae has been found to be structurally complex and unstable (e.g., one copy of the IR may be absent, major inversions sometimes exist; see [40,41,70]), the *O. basilaris* plastome was divided into the IR, LSC, and SSC (thus, the junctions of those three regions were excluded from the reference-guided assemblies), and those concatenated regions were used for reference-guided assembly. We aligned our plastome dataset using MAFFT in Geneious and then alignments were checked manually. A maximum likelihood (ML) analysis was carried out in Geneious with the RAxML [71] plugin, undertaking 1000 bootstrap (bs) pseudoreplicates using the GTR+gamma model of molecular evolution.

### 4.3. Divergence Time Estimation

We performed Bayesian divergence time estimation with MCMCTree [72] from the package PAML 4.9 [73]. First, the topology from the maximum likelihood inference (described above) was fixed and branch lengths were calculated under the GTR+G model with BASEML. Because fossils are not known in Cactaceae, we utilized three secondary calibrations from previously-inferred divergence time estimates across Cactaceae and Caryophyllales [19]: 29.904–40.096 MYA (millions of years ago) for the stem of Cactaceae, 24.876–32.324 MYA for the crown of Cactaceae, and 1.876–9.324 MYA for the crown of *Opuntia*. We maintained the default probability of 0.025 for violation of upper and lower bounds of secondary calibrations. We applied an independent-rates molecular clock with the birth-death model. Gamma priors were set to the default. We conducted three independent analyses, each with a different random seed, for 50 million generations, sampling every 100 generations. The first 25 million generations were discarded as burn-in. The summary output file was imported into Tracer v1.7.2 [74] to assess effective sample sizes (ESSs), considering ESSs greater than 200 adequate. The three runs yielded nearly identical results, indicating convergence, so the results from the first run were maintained and used for subsequent analysis. All divergence time estimation analyses were performed on University of Florida’s HiPerGator. Results were plotted with the R package MCMCtreeR [75].

### 4.4. Ancestral Area Estimation

We pruned the time-calibrated phylogeny for ancestral area reconstruction to include 20 taxa, 18 of which belong to the *Basilares* clade and two of which are representatives of its sister clade *Nopalea*, *O. guatemalensis* and *O. dejecta*. Two accessions of *O. polyacantha* var. *trichophora* were left in the analysis, because they represent distinct geographic distributions (Chihuahuan Desert vs. Colorado Plateau) and were non-sister taxa; however, *Hodgson 23,567* was removed, as that accession occupies the same distribution as *Majure 6079*. Nine areas were coded for the 20 taxa represented in the pruned phylogeny, constraining any node to have a maximum of three areas included in any ancestral state. In North America, coded areas followed Level III Ecoregions by the United States Environmental Protection Agency [76]. Biogeographic units were as follows: (A) AZ–NM Plateau, (B) Mojave Desert, (C) CA Pine Oak Mts, (D) CA Coastal Sage/Oak Woodlands, (E) Colorado Plateau, (F) Sonoran Desert, (G) Chihuahuan Desert, (H) Baja California, and (I) seasonally dry tropical forest of Central America and Mexico (SDTF). We coded the distribution for each taxon based on specimen data primarily from DES and the field experience of authors Hodgson, Majure, and Puente. Taxa distributed in seasonal tropical dry forests of Mexico and Central America were coded as a single area. Coding of SDTF as a single area is justified, because the aim was not to investigate finer-scale biogeographic history of *Opuntia* in South America. No dispersal constraints were applied.

We reconstructed ancestral areas under the following models, with and without the founder event speciation parameter (“jump” parameter or +J; [77]), totaling six models: dispersal–extinction cladogenesis (DEC; [78]), dispersal–vicariance analysis [79] with likelihood implementation (DIVALIKE), and a Bayesian approach with likelihood implementation (BAYAREALIKE; [80]). Log-likelihood (lnL) and the corrected Akaike information criterion (AIC) were calculated and used to select the best-fit model to our data.

All biogeographic analyses were performed in R 4.2.3 [81]. We used the R package BioGeoBEARS [77,82,83], with dependencies, cladoRcpp [84] and rexpokit [85,86,87], to conduct biogeographic analyses on the University of Florida’s Hipergator. Plotting of biogeographic results was performed with functions from the R package ape [88].

### 4.5. Ancestral Character State Reconstruction

To investigate morphological evolution in the Basilares clade, we conducted ancestral character state reconstruction for four characters: (1) epidermal pubescence (absence or presence); (2) cladode spine production (absence or presence); (3) fruit type (dry versus fleshy); and (4) pericarpel spine production (absence or presence). We used the Trace Character History Over Trees of Mesquite 3.6 [89] under the parsimony algorithm for taxa polymorphic for a given character state and under the ML reconstruction algorithm with the asymmetrical 2-parameter Markov k-state model (Mk1) for taxa with non-polymorphic states onto our single best ML tree.

## 5. Conclusions

The Xerocarpa clade originated in the Colorado Plateau, a cold desert, from where they proceeded to migrate southward back in to the Chihuahuan Desert, the AZ–NM Plateau, and then into the Mojave and Sonoran deserts, and the California montane vegetation zones. The Grand Canyon, given its immense size coupled with the hampered dispersal ability of dry-fruited Opuntias, likely played a major role in shaping the southern migration of the Xerocarpa clade out of the Colorado Plateau. Future studies should focus on organismal groups with similar distributions to test for a “Grand Canyon signal” in their biogeographic history.

## Figures and Tables

**Table 1:** Summary of model parameters and statistics. Parameters are dispersal (d), extinction (e), and jump (j). Summary statistics are log-likelihood (LnL) and corrected Akaike information criterion (AICc).

Model	Number of Parameters	d	e	j	LnL	AICc
DEC	2	0.03215266	4.375713 × 10^−2^	0	−55.27566	115.2572
DEC+J	3	0.01711820	1.000000 × 10^−12^	0.03940934	−50.40807	108.3161
DIVALIKE	2	0.03401536	2.001000 × 10^−9^	0	−52.35097	109.4078
DIVALIKE+J	3	0.02024158	1.000000 × 10^−12^	0.02959976	−49.65643	106.8129
BAYAREALIKE	2	0.03844895	3.691173 × 10^−1^	0	−58.96130	122.6285
BAYAREALIKE+J	3	0.01266913	4.732005 × 10^−2^	0.05175805	−53.18821	113.8764

## Data Availability

Our datasets and trees generated from this work are available on Figshare (10.6084/m9.figshare.23511972). Raw reads from genome skimming are available in the NCBI SRA database under project number PRJNA990351 (see Appendix A).

## Supplementary Materials

The following supporting information can be downloaded at: https://www.mdpi.com/article/10.3390/plants12142677/s1, Figure S1: DEC+J biogeographic analysis.

## Appendix A

Taxa sampled for our phylogenetic analyses, along with their collection or botanical garden accession numbers, herbarium repositories, and GenBank accession numbers. Data used from GenBank are given with their accession numbers. All new accessions sequenced here are available under Bioproject PRJNA990351.

***Consolea corallicola*** Majure 3321 (FLAS), SAMN15717090; ***Cylindropuntia bigelovii*** Majure 5496 (FLAS), SAMN12504902; ***Leptocereus sylvestris*** Majure 6874 (DES), SAMN16988921; ***Mammillaria heyderi*** Majure 7054 (DES), SAMN17293539; **Melocactus oreas** (SAMEA11053225); ***Opuntia arechavaletae*** Majure 6959 (DES, FLAS), SAMN12504939; ***Opuntia atrispina*** Majure 5636 (DES); **Opuntia austrina** Majure 4216 (FLAS), SAMN12504940; ***Opuntia basilaris* var. *basilaris*** L.C. Majure 5753 (DES); ***Opuntia basilaris* var. *brachyclada*** L.C. Majure 5744 (DES); ***Opuntia basilaris* var. *heilii*** L.C. Majure 6067 (DES); ***Opuntia basilaris* var. *longiareolata*** DBG 1952-3399-01 (DES), ***Opuntia basilaris* var. *ramosa*** M. Baker 18413 (ASU); ***Opuntia basilaris* var. *treleasii*** M. Baker 18541 (ASU); ***Opuntia chlorotica*** Majure 6132 (DES); ***Opuntia comonduensis*** DBG 2011-0082 (DES); ***Opuntia dejecta*** Majure 7064 (FLAS); ***Opuntia diploursina*** Stock 1869 (UT); ***Opuntia excelsa*** DBG 1986-0546 (DES); ***Opuntia guatemalensis*** Majure 7117 (FLAS); ***Opuntia macrorhiza*** Majure 5610 (DES); ***Opuntia microdasys*** DBG 1987-0449-21 (DES); **Opuntia pachyrrhiza** Puente 620 (ASU); ***Opuntia polyacantha* var. *arenaria*** M. Moore 2911 (FLAS), ***Opuntia polyacantha* var. *trichophora*** Majure 3526 (FLAS), W.C. Hodgson 23567 (DES), Majure 6079 (DES); ***Opuntia pycnantha*** DBG 1968 9278 (DES); ***Opuntia quitensis*** Majure 3848 (FLAS), SAMN12504941; ***Opuntia rufida*** Majure 5655 (DES); ***Opuntia stenopetala*** DBG 1984-0153-01 (DES); ***Opuntia tapona*** DBG 1939 0093 0101 [Gates s.n. (DES, FLAS)]; ***Pereskia grandifolia*** (SRR1698106); ***Pereskiopsis aquosa*** DBG 1997-0185 (DES), SAMN12504942; ***Portulaca oleracea*** Majure 6908 (FLAS); ***Tacinga palmadora*** Majure 8548 (FLAS), MT369930; ***Tephrocactus articulatus*** DBG 2001 0014 (DES), SAMN12504945.
