# Supplementary material for: Evolution of the Xerocarpa clade (Opuntia; Opuntieae): Evidence for the Role of the Grand Canyon in the Biogeographic History of the Iconic Beavertail Cactus and Relatives
Source: Plants (Basel). 2023 Jul 18;12(14):2677. doi: 10.3390/plants12142677 (PMC10385227; doi:10.3390/plants12142677)
Supplement: Supplementary file 1 [file plants-12-02677-s001.zip › plants-2479520-supplementary.pdf]

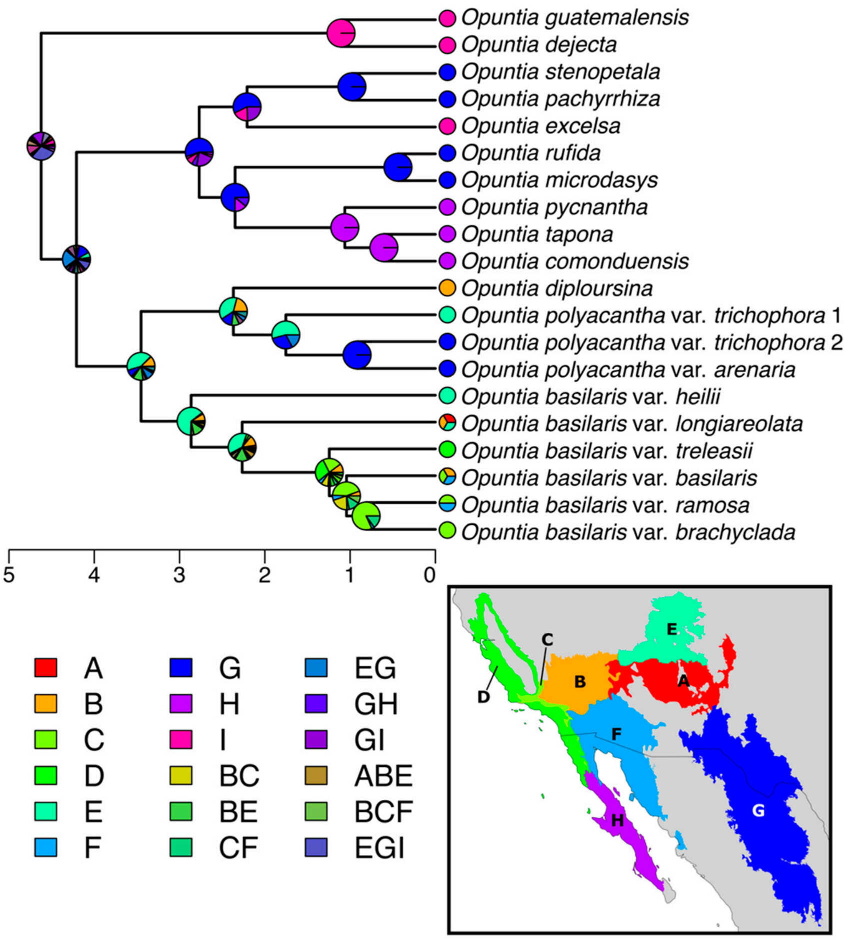

Figure S1. Ancestral area estimation based on the DEC+J model. The Xerocarpa clade evolved in the Colorado Plateau and moved south into the AZ/NM Plateau, the Mojave Desert and then into the Chihuahuan Desert in the Polyacantha clade and the Sonoran Desert and California montane forests in the Basilaris clade.
